# In the era of total mesorectal excision: adjuvant radiotherapy may be unnecessary for pT3N0 rectal cancer

**DOI:** 10.1186/1748-717X-9-159

**Published:** 2014-07-22

**Authors:** Jun-xin Wu, Yu Wang, Na Chen, Lu-chuan Chen, Peng-gang Bai, Jian-ji Pan

**Affiliations:** 1 Department of Radiation Oncology, Fujian Provincial Cancer Hospital, Provincial Clinical College of Fujian Medical University, Fuzhou, Fujian P R China

**Keywords:** Rectal cancer, Post-operative radiotherapy, Prognosis factors

## Abstract

**Background:**

Due to the Total Mesorectal Excision (TME) surgery made a good local control,the role of radiotherapy in the treatment of pT3N0 rectal cancer is debated and whether this group of patiens were overtreated has been a controversy recently. This study aimed to evaluate the value of adjuvant radiation after TME and survival outcome for patients with pT3N0 rectal adenocarcinoma.

**Methods:**

From January 2003 to December 2011, a total of 141 patients with pT3N0 rectal cancer after radical resection with the principle of Total Mesorectal Excision (TME) were enrolled. Among them, 42 patients (29.8%) got adjuvant chemotherapy (CT) and the remaining cohort received chemoradiotherapy (CRT). The 5-year overall survival rate (OS), 5-year disease free survival rate (DFS), 5-year local recurrence free survival rate (LRFS), 5-year local recurrence rate (LRR) and the prognostic factor of this cohort were analyzed.

**Results:**

The median follow-up interval time was 44 months. The 5-year OS and DFS rates were 82.4% and 71.9% for the whole group. There were no significant differences in 5-year OS (83.3% vs 72.4%, P = 0.931) or LRFS rates (81.7% vs 74.5%, P = 0.157) for patients between CT group and CRT group. Multivariate cox regression analysis suggests that preoperative serum CEA level, number of lymph nodes inspected, perirectal fat infiltration were independent prognostic factors for 5-year DFS. The recurrence rate was not affected by radiotherapy for patients with lower and midrectal cancer.

**Conclusions:**

For the patients with pT3N0 rectal cancer, addition radiation after TME surgery made no significant differences in survival rate and local recurrence rate. The effect of adjuvant radiotherapy needs further evaluation.

## Introduction

Rectal cancer is still a common tumor among the world,while the major treatment for patients with a rectal malignancy is radical surgical excision. For patients with resectable rectal cancer, an optimal surgery could get a satisfactory local control; while for those with advanced tumors, a curative resection may be especially difficult because of the special location of pelvis and always with high local failure rates [1]. As a result, a multidisciplinary synthetic therapy is of great important value. In the 1990s, several researches have suggested that postoperative chemoradiotherapy can cut down local recurrence rates [2,3]. So the US National Institutes for Health advocated that the postoperative chemoradiotherapy as standard regimen for patients with stage II or III rectal cancer [4]. Of note, all these previous researches were conducted in the times of conventional surgery method. More over,among the patients involved in previous studies, the respective proportion of patients with stage II and III remained unknown and the separate survival outcome of stage II rectal cancer were not illuminated too. Nowadays, radical radical surgery with the principle of TME (low anterior resection LAR or abdominalperineal resection APR) could lower the local recurrence rate significantly [5,6]. The TME has showed superior results than conventional surgery and has been the standard operation form for a resectable rectal cancer. With the low recurrence rate in the new times, the effect of conventional trimodality therapy (surgery plus chemotherapy plus radiotherapy) for stage II and III rectal cancer seems to be reconsidered. Consequently, the real benefit of addition radiotherapy after surgery was a question need to be investigated [7].

As mentioned above, for the pT3N0 rectal patients, several retrospective studies showed the low rates of local recurrence ranging from 4.1% to 6.5% after TME surgery alone [5,6]. What’s more, many studies have showed the addition of radiation therapy did not improve the survival outcome and local recurrence rate [7-9]. This result in favor of the point of view that for pT3N0 rectal cancer, routine adjuvant radiation therapy after TME may be unnecessary and overtreatment. Many researchers argue that the pT3N0 rectal cancer may have an “intermediate” risk of recurrence, and suggest that the radiation therapy need further investigated [10,11]. To evaluate the effect of adjuvant radiotherapy in patients with T3N0 rectal cancer after TME, we studied the influence of adjuvant radiotherapy on the survival and the recurrence rate among this cohort of patients.

## Materials and methods

### Patient selection

Patients with resectable adenocarcinoma and treated with TME at Fujian Provincial Cancer Hospital between January 2003 and December 2011 were collected. The patient selection criteria come as follows: (1) between 20–80 years old; (2) without neo-adjuvant chemotherapy and radiotherapy; (3) received radical resection with the principle of TME; (4) biopsy-proven pT3N0 rectal cancer according to the 7^th^ version of AJCC guideline; (5) no evidence of distant metastases and serious comorbidity before surgery; (6) received adjuvant chemotherapy (CT) after surgery. Ultimately, 141 patients were included in this study. Patients were split into CRT and CT group and evaluated for age, sex, pre-operative serum carcinoembryonic antigen (CEA) level, histologic differentiation, cell histology, grade of differentiation, total number of lymph nodes retrieved, lymphovascular invasion, adjuvant radiotherapy, development of recurrence or metastasis and survival. Before surgery, patients received a complete physical examination, blood routine test, liver and renal function test, proctoscopy, pelvic CT or MRI, chest X-ray and the level of CEA. Those who had a poor physical condition or metastasis were excluded from our study.

### Treatment

Surgery method: except the upper rectal cancer (10 cm above the anal verge), all patients got surgery with the principle of TME. The operation method was selected according to the digital rectal and proctoscopy examination. LAR was performed in 97 patients; the remaining 44 patients underwent APR. The surgery were operated by at least two of experienced surgeons. The hypogastric nerve, pelvic autonomic nerve plexus and arteria rectalis media were protected as far as possible, lymph drainage area and fat tissue were removed. The distal mesorectal margin was at least 2 cm away from the tumor, for those with a distance ranging from 1 to 2 cm from the anal verge; an intraoperative frozen pathological examination should be conducted to confirm the negative surgical margins.

Chemotherapy program: All of the patients received adjuvant chemotherapy or concurrent chemoradiotherapy. The 5-Fluorouracil (5-FU) -based chemotherapy program consisted of 2250 mg/m2 of 5-FU civ for 46 hours and 400 mg/m2 of CF for d1, and Oxaliplatin for d1 every 14 days for 8–12 cycles. In case of patients treated by oralling Xeloda, the patients took orally 1250 mg/m2 twice daily days 1-14 every 3 weeks to a total of 6 months. Concurrent chemotherapy: 225 mg/m2 of 5-Fu over 24 hours 5 days/week during radiotherapy or took Xeloda 825 mg/m2 twice daily 5 days per week during radiotherapy.

Radiation therapy: for the CRT group, the patients began additional radiation therapy within 4 weeks after TME. Of these patients, 27 got conventional radiotherapy,11 received three dimensional conformal radiation therapy, the remaining 4 received Intensity Modulated Radiation Therapy. A 6 MV dual photon linear accelerator was used to deliver the X-ray and three or five-field box technique was applied to the treatment planning. The radiation field was as follows: the upper bound was the level of L5-S1, the lower bound was the obturator formamen (Dixon) or 1.5 cm inferior of the metal sign (Mile’s), the lateral bound was 2.0 cm lateral to the widest bony margin of the true pelvis. A total dose of 50 Gy was delivered in 25 fractions of 2 Gy per day.

### Definition of treatment failure

Through reading the follow up data of the 141 patients, the treatment failures were found out. Local-failure was defined as any recurrence occured within the pelvis, including the tumor bed, regional lymph nodes, anastomosis, or perineal scar. While recurrence detected in the liver, lung, brain, and other organs or lymph nodes outside the pelvis were regarded as distant failure.

### Follow up

Patients were followed up routinely at 3-month intervals for the first 2 years, at 6-month intervals for the next 5 year, and once a year thereafter. The follow up examination consisted of a physical examination, measuring of CEA, chest X-rays, the whole body ECT, abdominal and pelvic CT or MRI. Survival time was calculated from the date of surgery to the date of die or loss to follow-up; local-recurrence free survival LRFS was from the date of surgery to the date of having local relapse; disease-free survival DFS was time from the date of surgery to the date of local recur, metastasis or die of the rectal cancer.

### Statistical analysis

SPSS 17.0 was used to analysis the data. 5-year OS, DFS and LRFS curves were calculated according to the Kaplan-Meier method and log rank test was used to distinguish the differences between groups. Chi-square tests was used to paired the clinicopathologic characteristics of two groups. The Cox regression was used for examining the independent factors associated with survival outcome. P < 0.05 was considered statistically significant.

## Results

### Clinicopathologic characteristics

Of the 141 patients, 42 received CRT, 99 accepted CT. All of the patients had a Karnofsky score higher than 90 point. Radiotherapy was performed significantly more commonly in those with lower rectal cancer (P = .003) and those with less number of lymph nodes (P = .001). The mean number of the lymph nodes was 16.15 (range, 0-50). In addition to above,the two groups were well matched in other clinicopathologic items such as age, sex, pre-operative level of CEA, histological type, grade of differentiation, maximum diameter of tumor, lymph-vascular invasion, perirectal fat infiltration (Table 1).

**Table 1:** Clinicopathologic characteristics of patients

**Characteristics**	**CT n = 99 (%)**	**CRT n = 42 (%)**	**P value**
Age, years	56	56.5	0.814
<50	31 (31)	14 (33)	
≥50	68 (69)	28 (67)	
Sex			0.966
Male	68 (69)	29 (69)	
Female	31 (31)	31 (31)	
Median Karnofsky scores	90	90	
Preoperative CEA (n g/m L)			0.088
<5	66 (67)	34 (81)	
≥5	33 (33)	8 (19)	
Distance to anal (cm)			0.003
0-5	34 (34)	27 (64)	
6-10	44 (44)	12 (29)	
11-15	21 (21)	3 (7)	
Surgical type			0.000
<Dixon	79 (80)	18 (43)	
Miles	20 (20)	24 (57)	
Histologic type			1.000
Adenocarcinoma	94 (95)	40 (95)	
Mucinous adenocarcinoma	5 (5)	2 (5)	
Grade of differentiated			0.665
Well	15 (15)	9 (21)	
Moderately	81 (82)	32 (76)	
Poor	3 (3)	1 (3)	
Maximum diameter (cm)			0.900
<5	53 (54)	22 (52)	
≥5	46 (46)	20 (48)	
Number of retrieved lymph nodes	37 (37)		0.001
<15	62 (63)	29 (69)	
≥15		13 (31)	
Lymph-vascular invasion			0.084
Yes	3 (3)	6 (14)	
No	96 (97)	36 (86)	
Perirectal fat infiltration			0.323
Yes	36 (36)	18 (43)	
No	63 (64)	24 (57)	

### Follow up and recurrence

The median follow up time was 44 months (range, 3-104 months). The proportion of patients followed up more than 60 and 36 months were 63.8% and 22%. During the follow up, a total of 23 patients (16.3%) relapsed. The 5 year cumulative recurrence rate was 8.2%. There was no significant difference in 5 year local recurrence rate between CT and CRT group (5.3% vs 14.3%, P = 0.140). Of the 23 recured patients, 11 patients suffered from local recurrence (7.8%), 17 patients had distant metastasis (12.1%), both local and distant failures happened to 5 patients (3.5%). Liver was the most common site of metastasis (39.1%), then was the lung (30.4%). Until the last follow up time, up to 20 patients died of rectal cancer, 1 patient died of other reasons. The median local recurrence and distant failure time were 44 months (range, 3–104 months) and 40 months (range, 3-104 months); The median survival time after local recurrence and distant metastasis were 17 months (0–49 months) and 21 months (0–49 months).

### Survival

**Figure 1:**
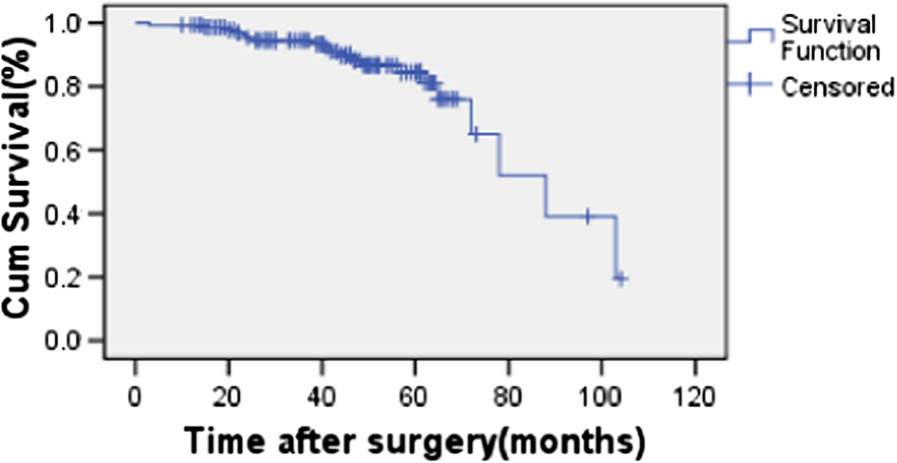
Overall survival of all patients.

**Figure 2:**
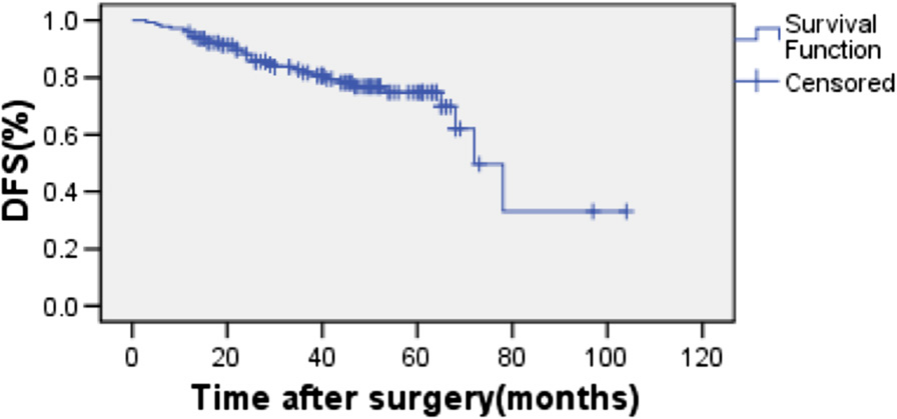
Disease-free survival of all patients.

**Figure 3:**
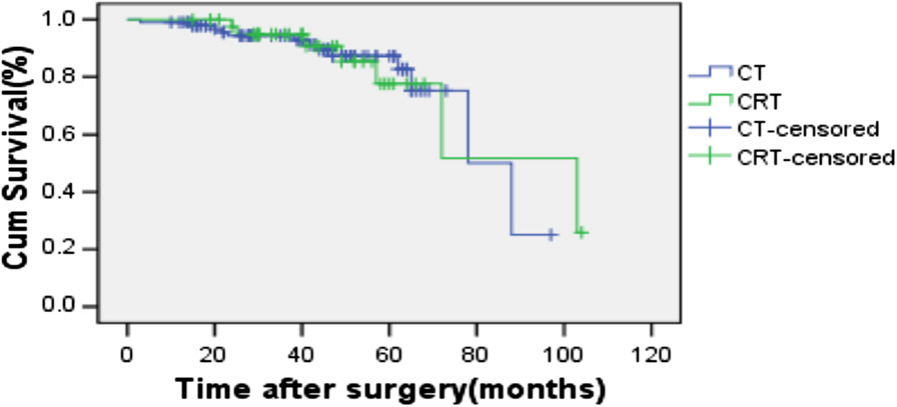
Overall survival rates with or without radiotherapy.

**Figure 4:**
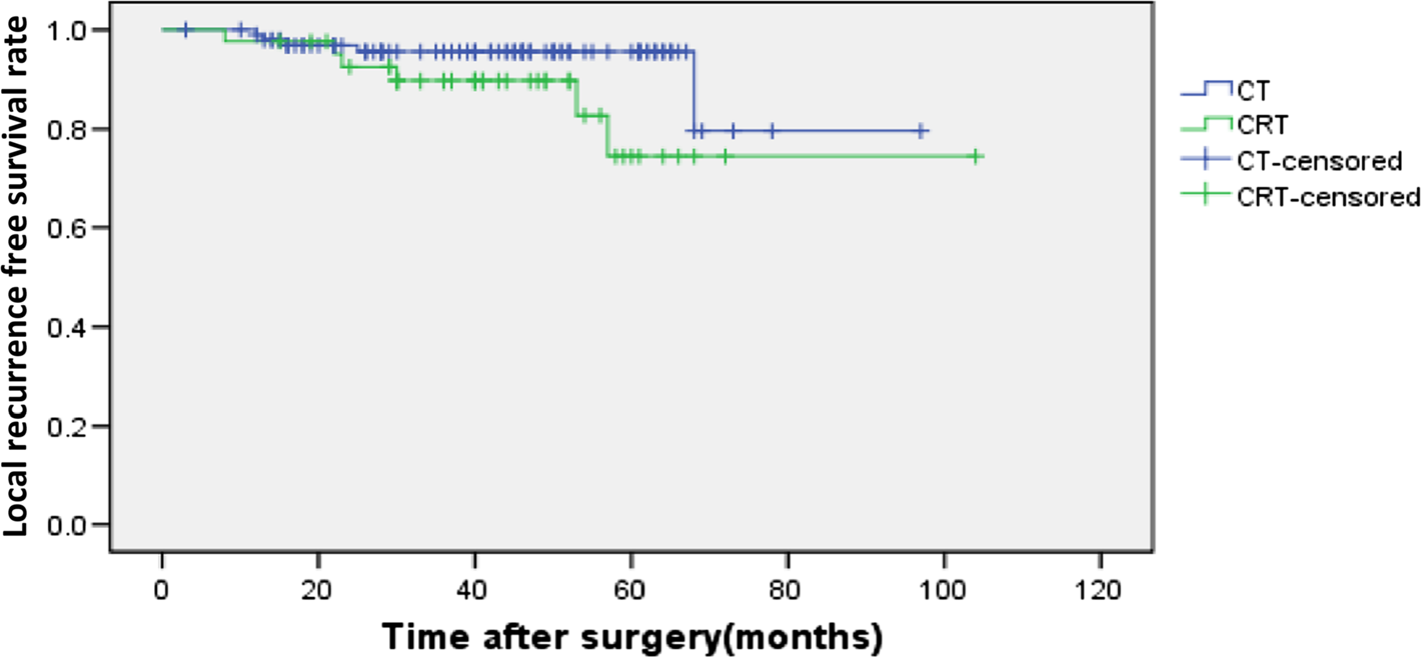
**Local-recurrence free survival rates with or without radiotherapy (N=141).** The difference of the overall survival between the two groups was not significant (P=0.157). CT chemotherapy, CRT chemoradiotherapy.

**Figure 5:**
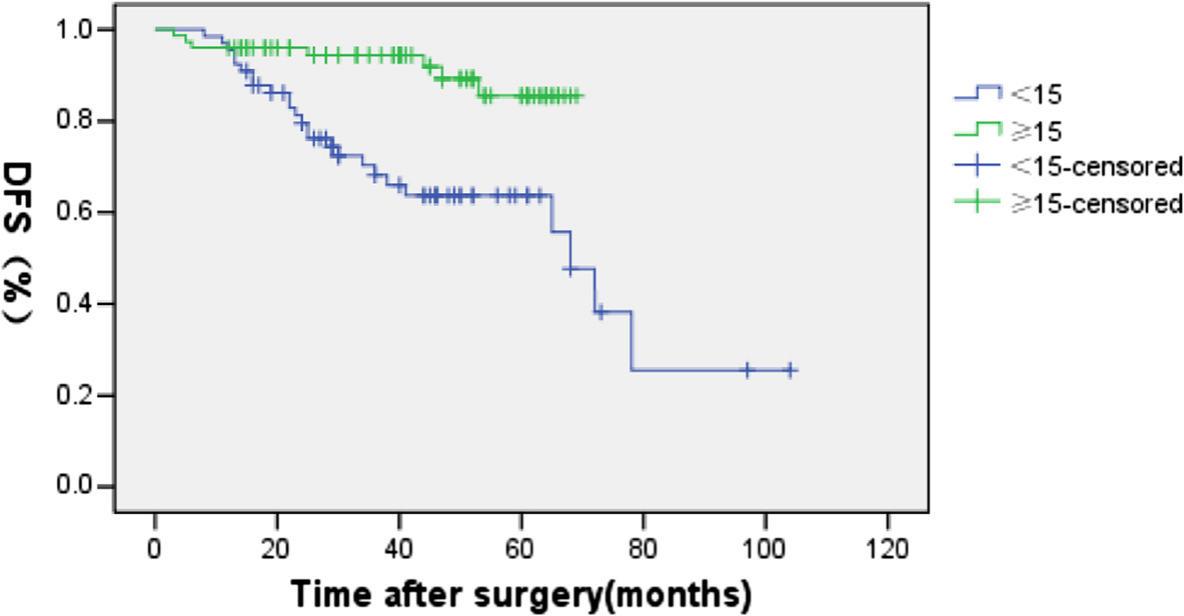
Disease-free survival rates with<15 and those ≥15 lymph nodes retrieved (P=0.001).

The overall 5-year OS and DFS rates were 82.4% and 71.9% for the whole group (Figure 1, Figure 2.). For the CT and CRT groups, the 5-year OS rates were 83.3% versus 72.4% (P = 0.931), respectively (Figure 3.), and the 5-year LRFS rates were 81.7% versus 74.5% (P = 0.157) (Figure 4). The 5-year DFS rate was significantly better in patients with ≤15 than in those with >15 examined lymph nodes (P = 0.001, Figure 5).

### Univariate analysis and cox regression

In the univariate analysis, preoperative level of CEA, the number of lymph nodes retrieved (<15 and ≥ 15), the differentiation of the tumor and the perirectal fat infiltration were significant prognostic factors affecting disease-free survival rate (Table 2). In the multivariate analysis, pre-operative level of CEA, number of lymph nodes and the perirectal fat infiltration were independent factors associated with significantly worse DFS (Table 3).

**Table 2:** The log-rank univariate analysis of patients

**Factors**	**Number**	**3-year DFS (%)**	**5-year DFS (%)**	***χ***^**2**^	**P value**
Age (year)				2.104	0.14
<50	45	85.4	76.9		
≥50	96	78.8	55.4		
Sex				1.208	0.272
Male	97	80.3	68.5		
Female	44	83.7	69.7	6.563	0.010
Pre-operative CEA (n g/m L)					
<5	100	87.4	74.1		
≥5	41	65.1	52.7		
Distance to anal (cm)				3.299	0.19
0-5	61	80.3	68.0		
6-10	56	79.9	61.8		
10-15	24				
Operation type				1.958	0.162
Dixon	97	85.8	74.1		
Miles	44	71.5	58.1		
Histologic type				3.053	0.081
Adenocarcinoma	134	83.1			
Mucinous adenocarcinoma	7	44.8			
Grade of differentiated				29.906	0.000
Well	24	80.8	68		
Moderately	113	83.5	68		
Poor	4	4.7			
Maximum diameter (cm)				0.106	0.74
<5	75	80.5	59.5		
≥5	66	81.9	70.6		
Number of retrieved lymph nodes				11.616	0.000
<5	66	68.2	57.4		
≥5	75	93			
Lymph-vascular invasion				0.087	0.768
Yes	9	85.7	22.4		
No	132	81.6	71.9		
Radiation				2.787	0.095
Yes	42	87.9	74.5		
No	97	91.2	90.7		
Perirectal fat infiltration				3.953	0.009
Yes	55	76.2	40.5		
No	86	86.5	71.9		

DFS: disease free survival; LRR: local recurrence rate.

**Table 3:** The multivariable analysis of patients

**Factors**		**β value**	**SE value**	***χ***^**2**^	**P value**	**95% CI**
	Pre-operative CEA					
	(<5 ng/m L, ≥ 5 ng/m L)	1.271	0.420	9.136	0.003	1.563,8.120
DFS	Number of lymph nodes						
	(<15, ≥ 15)	−1.774	0.553	10.280	0.001	0.057,0.502	
	Perirectal fat infiltration						
	(Yes/No)	0.860	0.431	3.981	0.046	1.015,5.501	
DFS	Number of lymph nodes	−1.600	0.787	4.129	0.042	0.043,0.945	
	inspected (<15, ≥ 15)						
	Perirectal fat infiltration	1.617	0.700	5.338	0.021	1.278,19.865	
	(Yes/No)						

### Effect of radiotherapy on recurrence

Patients with mid to lower rectal cancer had a higher local recurrence rate than those with upper rectal cancer, while the difference was not significant (P = 0.115). No matter of the height of rectal cancer, radiation did not make significant improvement in local recurrence rate (Table 4). Similarly, local recurrence rate was also higher in patients with ≤15 (13.6%) than in those with >15 (2.7%) examined lymph nodes (P = .015). According to different number of lymph nodes,the recurrence rate differed not significantly between the CT and CRT groups (Table 5).

**Table 4:** The effect of radiotherapy on recurrence in different height of rectal cancer

**Distance**	**Recurrence rate (%)**	**P value**	**CT**	**CRT**	**P value**
0-5 cm	9.8	0.115	2/34 (5.9%)	4/27 (14.8%)	0.392
6-10 cm	8.9	—	3/44 (6.8%)	2/12 (16.7%)	0.289
11-15 cm	0	—	0/21 (0%)	0/3 (0%)	—

**Table 5:** The effect of radiotherapy on recurrence in different number of lymph nodes

**Number**	**Recurrence rate (%)**	**P value**	**CT**	**CRT**	**P value**
<15	13.6	0.115	4/37 (10.8%)	5/29 (17.2%)	0.693
≥15	2.7	—	1/62 (1.6%)	1/13 (7.7%)	0.319

## Discussion

Many studies showed the post-operative radiation could decrease the rate of local relapse and improve survival rate for stage II and III rectal cancer. While, with the introduction and application of TME in rectal cancer which significantly reduced the recurrence rate of resectable rectal cancer, the routine postoperative radiation may not be necessary, especially for subgroup patients of pT3N0 rectal cancer, with an “intermediate” risk for recurrence. Oppositely, the new role of preoperative RT has been confirmed for stage II or III rectal cancers. The MRC CR07/NCIC-CTGC016 conducted a multicenter randomized trial comparing preoperative radiotherapy with selective postoperative chemoradiotherapy. The median follow-up time was 4 years and it came to a result that preoperative radiotherapy significantly improved the 5-year DFS and reduced local recurrence rate [12]. To evaluate the value of pre-operative radiation for patients with resectable rectal cancer who undergone TME, a prospective randomized trial contained of 1861 was conducted. By comparing the outcome between the TME group and pre-operative 5*5 Gy short-term radiation plus TME group, the study showed that pre-operative radiotherapy could significantly reduce the local recurrence rate. The rate was 5.6% in pre-operative radiation group while the TME group was 10.9% (P < 0.001) [13]. In the study of Kapiteijn [14], the local recurrence rate at two years was 2.4% in the pre-operative radiation plus TME group and 8.2 percent in the TME group (P < 0.001), confirming that for patients received TME, short-term preoperative radiotherapy reduces the risk of local recurrence. As a result, many researchers suggest pre-operative radiation has more advantage over post-operative radiation. Because of the low local recurrence rate after an optimal TME surgery, the risk of additional radiation in patients with T3N0 rectal cancer may outweigh the potential advantages. Nissan et al. [6] reported patients with pT3N0 rectal cancer had a 4.1% local recurrence rate and 71.4% overall survival with TME alone,and the local and distant RFS and DSS were similar with pT2N0 rectal cancer. According to the report of Merchant NB [15], the overall local recurrence was 9% and overall survival was 75% for patients with T3N0 rectal cancer who underwent surgery without adjuvant treatment. Also, many researches showed additional postoperative radiotherapy did not alter local recurrence or survival after TME in patients with stage IIA rectal cancer [8,9]. They proposed addition postoperative radiation may be overtreatment for patients with stage II A rectal cancer if they had no other risk factors. A study conducted by Gunderson showed that patients with T3N0 rectal cancer had similar prognosis with T1/2 N1 rectal cancer,and the 5-year OS and DFS were 84% and 69% after surgery plus chemotherapy for this subgroup, and adding radiotherapy did not improve the survival, suggesting that a trimodality treatment approach was unnecessary [16]. Moreover, the morbidities and poor functional outcomes induced by radiotherapy always affect the life quality of patients. Ooi reported that for rectal patients receiving postoperative radiotherapy, the acute toxicities incidence rate is ranging from 4%-48% and 3%–10% of cases need hospitalization care [17]. In the long-term, radiation associated morbidities and dysfunctional outcomes such as fibrosis, autonomic nerve injury, bladder and sexual dysfunction were more common in patients receiving pelvic radiation compared to those without radiation [18-21]. Thus, for the patients with pT3N0 rectal cancer, the role of radiotherapy needs to be carefully evaluated.

In our retrospective analysis of 141 patients with T3N0 rectal cancer,we found that the 5-year OS and LRFS were not significantly different between CT and CRT group (Figure 3, Figure 4). The 5-year OS, DFS and local recurrence rate of the whole group were 82.4%, 71.9% and 7.8%, respectively; similar with the previous reports [7-9,16,22,23]. In terms of the effect of radiation on local recurrence rate,we found that no matter the number of lymph nodes,there was no significant difference between the CT and CRT groups. For the different height of the rectal cancer, radiation did not affect the recurrence rate of them either.

The risk factors that associated with the local recurrence have been inconsistently reported in many studies. In the study made by Nissan et al. [6], univariate analysis showed the presence of LVI, abnormal preoperative CEA, and older age were associated with pelvic recurrence, while the multivariate analysis showed only abnormal preoperative CEA was independent factors for DFS (RR =3.1; 95% confidence interval [CI], 1.2-8.1; P = 0.01) and DSS(RR =2.9, 95% CI: 1.1-7.6, P = 0.02). The involvement of the circumferential resection margin and age >60 years were also reported associated with adverse oncologic outcomes [9]. Merchant et al.showed the presence of LVI was significantly a predicting factor for local recurrence [14]. Willett et al. reported that the depth of perirectal fat invasion by the tumor was an independent factor affecting local recurrence [23]. The study of Tepper et al. showed the number of lymph nodes inspected and the lower rectal cancer were risk factors for local recurrence [24,25]. Recently, a prospective study using data from the MRC CR07 and NCIC-CTG CO16 randomized clinical trial showed that a negative circumferential resection margin and a superior plane of surgery were associated with low local recurrence rates, while a superior plane of surgery was an independent factor affecting the local recurrence rates [26]. In our study, we found that the pre-operative level of CEA and the number of lymph nodes inspected and perirectal fat infiltration were the independent factors affecting the 5-year DFS and local recurrence rate. The 5-year DFS was better and the recurrence rate was lower in patients with ≥15 than those with < 15 lymph nodes. According to a research by Swanson, the 5-year relative survival rate for T3N0M0 colon cancer was 64% if 1 or 2 lymph nodes were examined and rose to 86% if 25 lymph nodes were examined, the prognosis of T3N0 colon cancer is dependent on the number of lymph nodes examined [27]. Vather et al. also reported for Stage II and III colonic cancer, lower 5-year mortality was associated with increasing rates of nodal examination [28]. In the previous studies, upper rectal cancers have lower local recurrence rates than mid to lower rectal cancer [29-31]. In our study, we also found that upper rectal cancers have lower recurrence rates than mid to lower rectal cancer, though the difference was not significant (P = .115). This negative result may due to the small number of patients in our study and increasing the cohort may make it significantly different.

This study has several limitations as follows. Firstly, the 5-year OS and LRFS of CRT group were lower than those of CT group. This may partially caused by our selection bias, the patients got adjuvant radiotherapy have more adverse factors such as lower tumor location(P = .003) and less number of lymph nodes inspected (P = .001). Secondly, our study is a retrospective and nonrandomized analysis with a small sample size, this may not be convincing enough to prove the negative role of additional radiation after TME surgery for patients with no risk factors. Similarly, the little number of sample limits us to demonstrate the positive role for the pT3N0 patients with risk factors. However,we came to a result that was similar to the previous studies which sample sizes were larger than us. Thirdly, all of patients in our study were with negative circumferential resection margins which limit us to clarify the prognostic of patients with positive CRM. Lastly, with respect to location, the local recurrence rate was not of significant difference. A study with large sample will be needed to investigate all of these problems.

## Conclusion

Despite of limitations, we showed the additional radiotherapy did not significantly improve the overall survival and local recurrence rate in patients with pT3N0 rectal cancer. The recurrence rate is quite low for patients in CT group. If an optimal TME surgery operated by experienced surgeons guarantee a low local recurrence rate, the risk of post-operative recurrence can be improved through postoperative CT for rectal cancer patients with few risk factors. For these subgroup patients, adjuvant pelvic radiation after TME needs to be further evaluation. A prospective study is needed to confirm this conclusion.

## Competing interests

The authors declare that they have no competing interests.

## Authors’ contributions

JXW designed the study, and YW performed statistical analyses and wrote the manuscript. JXW conceived of the study. NC, LCC, PGB participated in the clinical coordination and revised the manuscript. All authors read and approved the final manuscript.
